# Commentary: harnessing the first peptidic modulator of the estrogen receptor GPER

**DOI:** 10.3389/fphar.2024.1413058

**Published:** 2024-05-01

**Authors:** Rosamaria Lappano, Marcello Maggiolini, Christophe Mallet, Yves Jacquot

**Affiliations:** ^1^ Department of Pharmacy, Health and Nutritional Sciences, University of Calabria, Arcavacata di Rende, Italy; ^2^Institut National de la Santé et de la Recherche Médicale (INSERM), NEURO-DOL Basics and Clinical Pharmacology of Pain, Université Clermont Auvergne, Clermont-Ferrand, France; ^3^ Faculty of Medicine, ANALGESIA Institute, Clermont-Ferrand, France; ^4^ Faculty of Pharmacy of Paris, Cibles Thérapeutiques et Conception de Médicaments (CiTCoM), Centre National de la Recherche Scientifique (CNRS) UMR 8038, INSERM U1268, Université Paris Cité, Paris, France

**Keywords:** peptide, modulator, GPER, nociception, breast cancer cells

## 1 Introduction

According to the pioneering work of Thomas et al., the G protein-coupled estrogen receptor GPER, a class A (rhodopsin-like) GPCR, interacts with estradiol (Thomas et al., 2005). This membrane protein is involved in a panel of pathophysiological actions including cardiovascular and digestive diseases (DeLeon et al., 2020; Groban et al., 2020), immune response (Notas et al., 2021), metabolic disorders (Sharma et al., 2018), neuroprotection (Pemberton et al., 2022) and cancer such as triple negative breast cancer (Zhang et al., 2024). Therefore, GPER has generated increasing attention in the scientific community and the synthesis of GPER modulators could open promising perspectives for the treatment of various diseases.

Recently, we have read with great interest the article published by E. Prossnitz and M. Barton entitled “The G protein-coupled estrogen receptor GPER in health and disease: an update” (Prossnitz and Barton, 2023). Although interesting, we consider that this article would benefit from our contribution to this fascinating field regarding the discovery of the first peptidic GPER modulator named ERα17p.

## 2 From the discovery to the anti-proliferative, anti-nociceptive and anti-inflammatory actions of ERα17p

The discovery of the peptide ERα17p and the assessment of its action through GPER is issued from European collaborations comprising research teams from Belgium, France, Greece and Italy. ERα17p corresponds to the residues 295-311 (hinge/AF2 region) and 123-139 of the human estrogen receptor α (ERα) and its isoform ERα36, respectively (primary sequence: PLMIKRSKKNSLALSLT).

In ERα- and GPER-positive ELT3 rat leiomyoma cells cultured under steroid-deprived conditions, ERα17p prompted proliferative activity through ERα, GPER, Gα_i_, EGFR, ERK1/2 and the translocation of β-arrestin. This effect being abolished by the GPER antagonist G-15 and a GPER siRNA, thus it occurred in a GPER-dependent manner (Leiber et al., 2015). In serum-cultured ERα-negative and GPER-positive MDA-MB-231 and SKBr3 human breast cancer cells, ERα17p was responsible for membrane-initiated molecular events leading to apoptosis *in vitro* and *in vivo* and, in the case of MDA-MB-231 cells, to the inhibition of migration (Kampa et al., 2011; Pelekanou et al., 2011). In similar conditions, ERα17p displayed anti-proliferative effects that were rescued by the selective GPER antagonist G-36, suggesting an inverse agonist action (Lappano et al., 2019). In MDA-MB-231 triple negative breast cancer cells, which were engineered to knock out GPER expression by CRISPR/Cas9 genome editing technology, ERα17p failed to show anti-proliferative effects, in contrast to that observed in wild type MDA-MB-231 cells (Jouffre et al., 2023). The abovementioned responses were initiated at the cell membrane (Kampa et al., 2011; Leiber et al., 2015; Pelekanou et al., 2011; Lappano et al., 2019). In addition, we have shown that in the presence of ERα17p, GPER becomes inactive and is degraded through the proteasome system, then resulting in a decrease of pEGFR, pERK1/2 and c-fos levels (Lappano et al., 2019). Accordingly, ERα17p decreases by about 50% the size of triple negative breast tumors xenografted in BalbC^−/−^ nude mice, at the dose of 1.5 mg/kg body weight, three times per week during 4 weeks (Pelekanou et al., 2011).

In addition to these antitumor actions, the peptide ERα17p has demonstrated GPER-dependent anti-nociceptive effects at the supraspinal level and anti-inflammatory activities, from 2.5 mg/kg, in inflammation animal models (Mallet et al., 2021; Jouffre et al., 2023).

Supporting previous data, a specific GPER antibody concomitantly used with a fluorescein-labeled version of ERα17p revealed superimposed fluorescence signals in SKBr3, therefore indicating a physical interaction between ERα17p and GPER (Lappano et al., 2019). In this regard, it is worth noting that ERα17p shares structural analogies with PBX1, a pyrrolobenzoxazinone acting as a GPER antagonist (Maggiolini et al., 2015; Lappano et al., 2019). Moreover, docking studies showed that ERα17p interacts in the low micromolar range through its N-terminal PLMI motif with the same extracellular GPER pocket, and more specifically through hydrogen and hydrophobic contacts with the residues Gln-138, Pro-192 and Ala-209, as displayed by other ligands (Lappano et al., 2019; Kampa et al., 2023). These observations suggested that the PLMI motif could play an important role in driving the action of ERα17p (Leiber et al., 2015; Lappano et al., 2019; Jouffre et al., 2023). Accordingly, the PLMI peptide displays similar effects as ERα17p. These results highlight also that the 295-311 and 123–139 sequences or, at least, their PLMI motif, may participate to the physical interaction of ERα and ERα36, respectively, with GPER (Acramel and Jacquot, 2022).

## 3 Conclusion

We believe that the interesting and timely issues raised by the article of E. Prossnitz and M. Barton would benefit from the addition of recent findings, in particular with respect to the list of GPER modulators including ERα17p and PLMI, as well as others, namely aldosterone, the diphenylacrylamide derivative STX and, possibly, the amyloid β1-42 peptide (Evans, 2019), and related physiological roles resulting from membrane-initiated signaling. A comprehensive review recapitulating the aforementioned data has been recently published (Kampa et al., 2023). Overall, we would like to highlight the relevance of considering this peptide in the list of GPER modulators.
